# Supplementary material for: Association between oral microbiome and seven types of cancers in East Asian population: a two-sample Mendelian randomization analysis
Source: Front Mol Biosci. 2023 Nov 21;10:1327893. doi: 10.3389/fmolb.2023.1327893 (PMC10702768; doi:10.3389/fmolb.2023.1327893)
Supplement: Supplementary file 1 [file DataSheet1.ZIP › Supplementary Materials/MR plots of saliva/saliva═╝/Prostate cancer/pheno.2188_to_prostate cancer_leave_one_out.pdf]

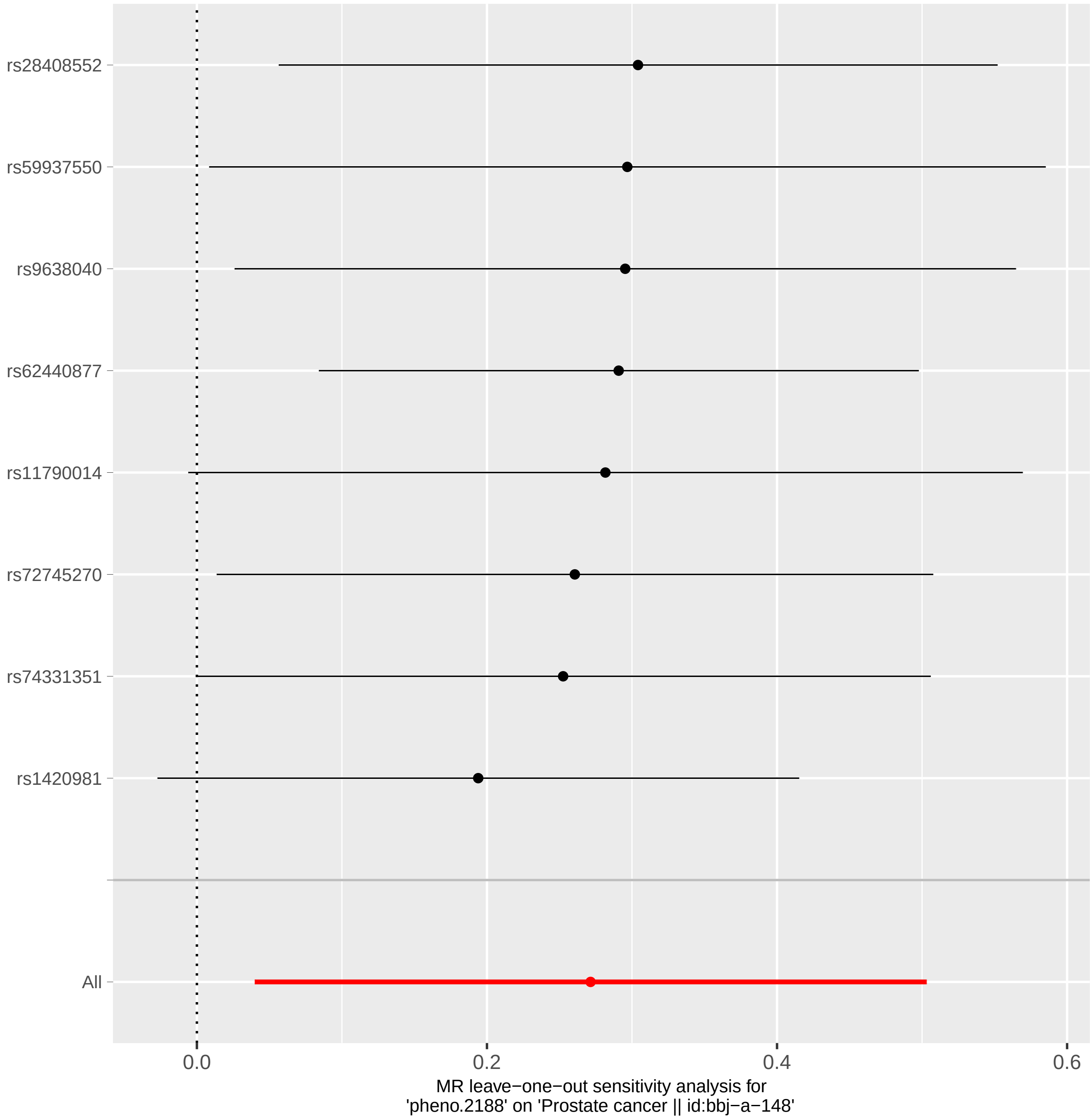
rs28408552
rs59937550
rs9638040
rs62440877
rs11790014
rs72745270
rs74331351
rs1420981
All
0.0
0.2
0.4
0.6
MR leave−one−out sensitivity analysis for
'pheno.2188' on 'Prostate cancer || id:bbj−a−148'
